# A Case of Histoplasma duboisii Brain Abscess and Review of the Literature

**DOI:** 10.7759/cureus.6984

**Published:** 2020-02-13

**Authors:** Landry Konan, Landry Drogba, Doukoure Brahima, Fassil B Mesfin

**Affiliations:** 1 Anatomy and Neurosurgery, University Felix Houphouet Boigny, Abidjan, CIV; 2 Neurosurgery, School of Medicine at the University of Abidjan, Abidjan, CIV; 3 Pathology, School of Medicine at the University of Abidjan, Abidjan, CIV; 4 Neurosurgery, University of Missouri, Columbia, USA

**Keywords:** brain abscess, histoplasma duboisii, histoplasmosis, mycotic disease, histoplasma capsulatum, capsulatum, duboisii

## Abstract

Histoplasmosis is a fungal disease caused by *Histoplasma capsulatum *var. *capsulatum *(Hcc) and *H. capsulatum *var. *duboisii *(Hcd)*. *Central nervous system (CNS) involvement is rare. So far, the few cases reported having Histoplasmosis associated brain abscesses were caused by *H. capsulatum *var. *capsulatum*. Herein, we report a unique case of brain abscess caused by *H. capsulatum* var. *duboisii *occurring in a 42-year-old immunocompromised woman with HIV. Initially, she presented with hypothermia, vomiting, frontal headache, evolving over one month. She then progressed to have a generalized seizure. Brain MRI showed multifocal brain abscesses and a frontal osteitis. The frontal osteitis was biopsied and confirmed the diagnosis of *H. capsulatum* var. *duboisii*. She was successfully treated with liposomal amphotericin B (150 mg daily) for the first four weeks and itraconazole (200mg twice daily) for six months.

## Introduction

Histoplasmosis is a fungal disease caused by *Histoplasma capsulatum* var. *capsulatum *(Hcc) and *H. capsulatum* var. *duboisii *(Hcd)*. *While the variant *capsulatum *is prevalent in the American continent, the variant *duboisii *is commonly reported endemically in western and Central Africa​​​​. Central nervous system (CNS) involvement is rare, and when it occurs, its typical presentation is meningitis [1-2]. So far, few cases of Histoplasmosis associated brain abscesses have been reported; they were all caused by *H. capsulatum* var. *capsulatum.* Herein, we reported a unique case of brain abscess by *H. capsulatum *var*. duboisii* occurring in an immunocompromised patient with HIV. 

## Case presentation

A 42-year-old woman, a poultry-seller, was admitted to our institution for hypothermia, vomiting, frontal headache, evolving over one month. Generalized seizures complicated the symptoms three days before admission. The patient has a history of Hepatitis B and has been positive for HIV1 for five years but voluntarily stopped her antiretroviral treatment. Physical examination showed that she was conscious, emaciated, had oral candidiasis, and a frontal scalp swelling. No neurological deficits were noticed. Laboratory results showed anemia (Hemoglobin = 10.5g/dl), lymphopenia (Lymphocyte count = 2.25*10^3^/µL) and a low CD4+ count of about 73 cells/mm^3^. Tuberculosis screening by bronchopulmonary sputum, gastric sampling analysis, and chest X-ray was unremarkable.

**Figure 1:**
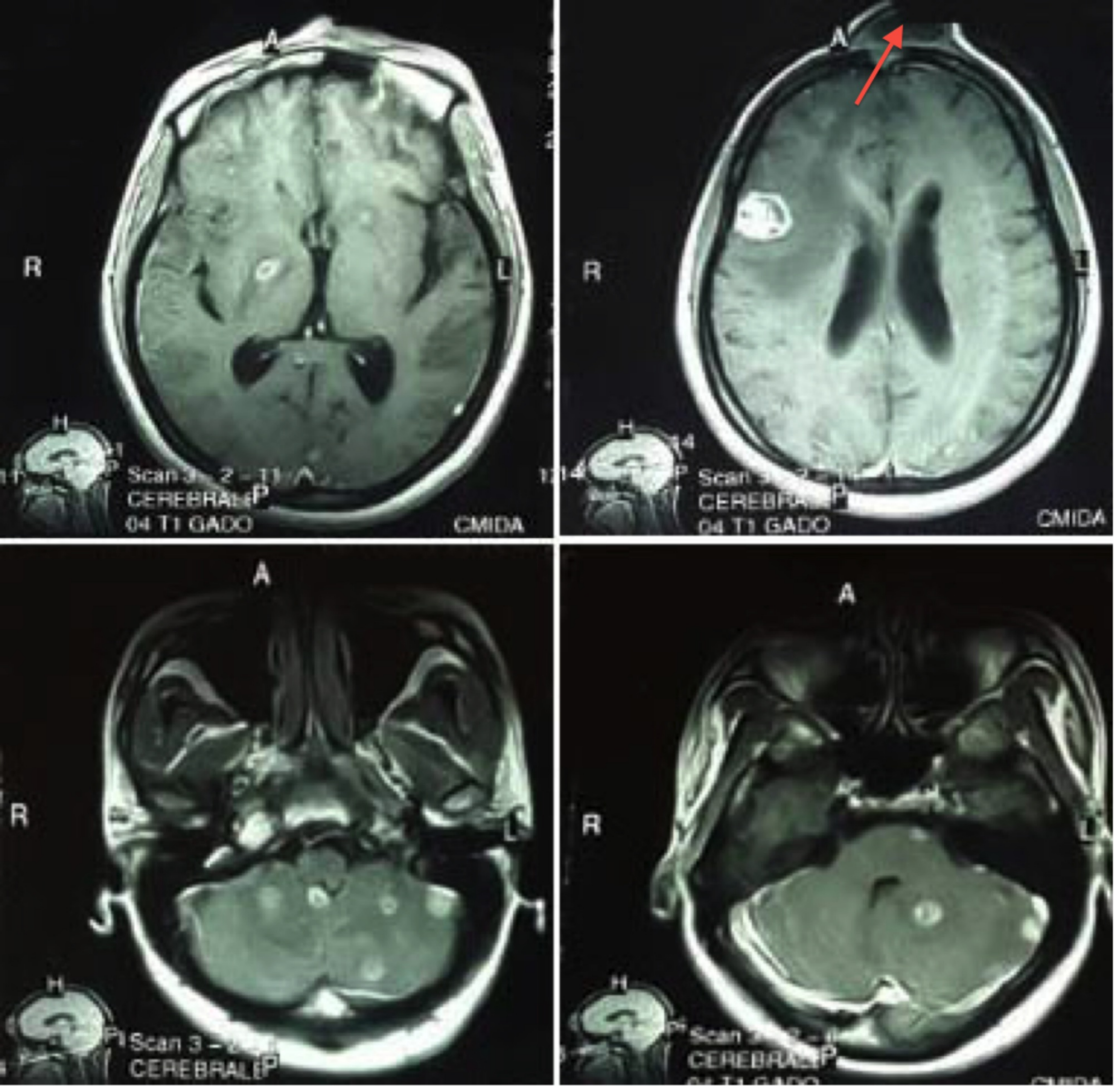
Gadolinium-enhanced MRI of the brain showing supra- and infratentorial round-shaped enhancing lesions and osteitis (red arrow)

**Figure 2:**
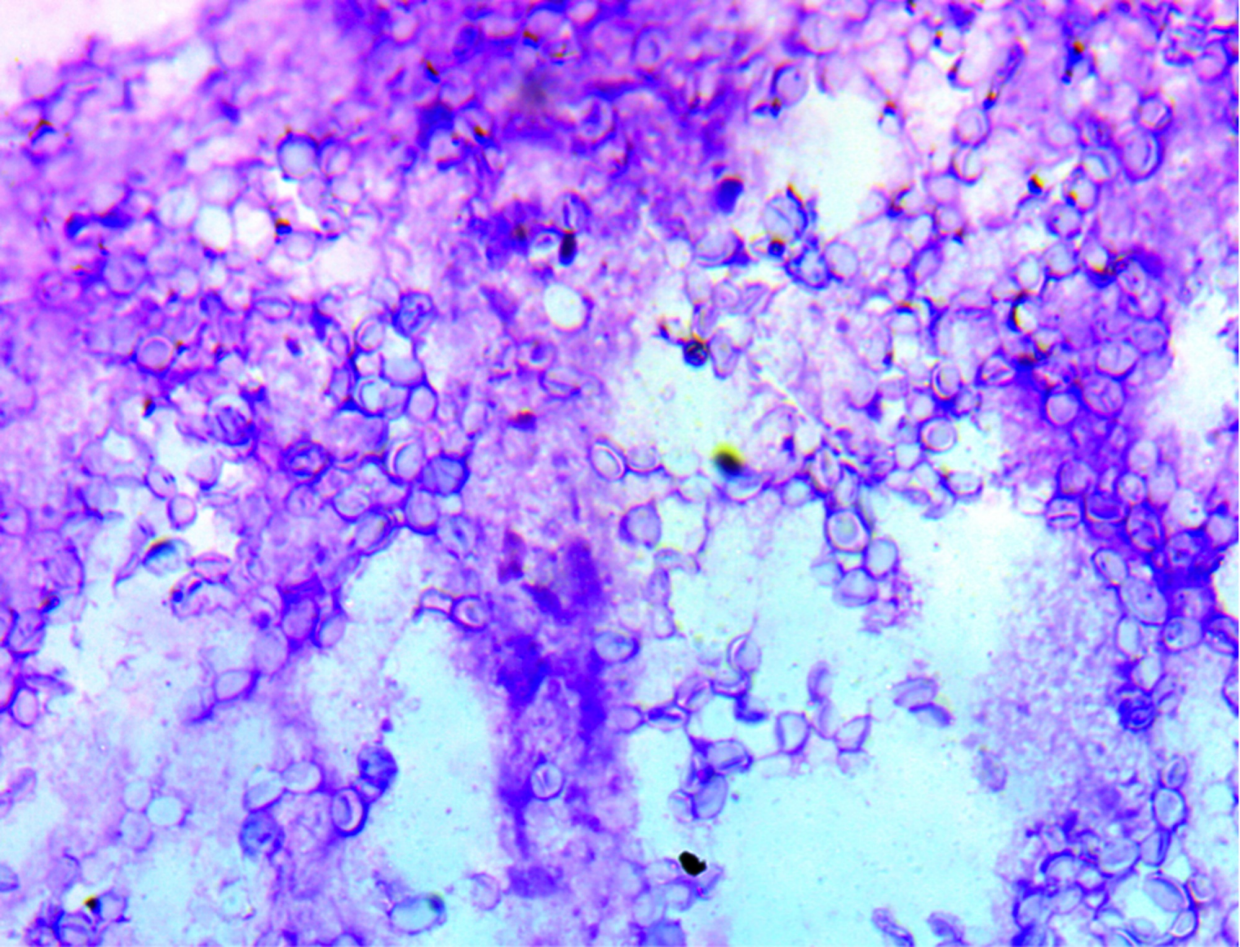
Direct examination of histoplasmosis (H. capsulatum var. duboisii) showing numerous intracellular yeasts with thick walls (May-Grunwald Gremus (MGG), x400)

A brain CT scan and MRI identified multifocal brain abscesses (frontal, cerebral, and cerebellar) associated with a frontal osteitis (Figure 1). The biopsy of the frontal lesion revealed a dark purulent fluid. Direct mycological analysis displayed several fungi with specific features evoking the diagnosis of *Histoplasma capsulatum *var. *duboisii *(Figure 2)*.*

The patient received an initial infusion of liposomal amphotericin B (150 mg daily) for the first four weeks of treatment. She started a new antiretroviral therapy. Subsequently, she was discharged with an oral dose of itraconazole (200mg twice daily) for six months. On the follow-up, eight months later, the patient was fine, and the post-therapeutic brain MRI and CT scan showed a resolution of brain abscesses. Consecutive follow-up examinations were scheduled every two months for one year. 

## Discussion

Species

Histoplasmosis is a fungal infection caused by two human pathogenic variants of *Histoplasma capsulatum: *var. c*apsulatum* and var. *duboisii*. A third pathogenic variant has been described in the equine population. Since the initial description in 1952 by Dubois, the infection by the variant* duboisii *has been referred to as the African Histoplasmosis [3-6]. The yeast is found in soil and materials contaminated with bird or bat droppings. The reservoir of *H. capsulatum* var. *duboisii*​​​​ is deemed to be in Africa as the published cases were reported from living in this continent, especially in West and Central Africa. The cases published from Spain and Japan were of African immigrants [6]. One exceptional case has been published in an autochthonous patient in India [4]. The disease is probably under-reported in Africa, where less than 300 cases have been published [5,6]. In the Ivory Coast, this is the sixth reported case, and to the best of our knowledge, this is the first African case of brain abscess [7].

Pathogeny

Although from the same species, the presentation and pathology of variants *duboisii *and *capsulatum* are variable. The variant* capsulatum* is endemic, not only in the United States around the Ohio and Mississippi River valleys but throughout the world, whereas the variant *duboisii *is limited to Africa [2]. On microscopy, the yeast cell sizes of variant *duboisii* are bigger, measuring 10-15 µm versus 2-5 µm for variant *capsulatum* [4,6]. Furthermore, the variant *capsulatum* is airborne, causing pulmonary infections, whereas the variant *duboisii *is involved in skin and bone infections [8]. The mechanism of the hematogenous dissemination of the infection to the meninges or brain is not well understood [9]. Some authors suggest that following inhalation of spores, the yeast forms thrive in the lungs and are initially controlled by helper T cells. In the case of T-cell immunodeficiency, the micro-organisms subsequently spread throughout the body via macrophages.

Clinical presentation

Published cases of CNS histoplasmosis were either meningitis, myelopathy, encephalitis, or focal parenchymal brain and spinal infections [9,10]. Some cases of stroke by infectious emboli and ventriculoperitoneal (VP) shunt infection have also been reported. CNS histoplasmosis occurs in 5-20% of disseminated infection, where T-cell immunodeficiency is a critical predisposing factor [10,11]. In our case, the patient was a poultry worker with untreated HIV infection. Therefore, her work environment, along with the HIV condition, made her be a typical vulnerable subject for CNS histoplasmosis. Multifocal supratentorial and infratentorial brain abscesses have been described in AIDS patients. However, those cases only involved the variant* capsulatum * [10-12].

African histoplasmosis frequently affects bony structures such as the vertebrae, skull, femur, and humerus. In our case, the patient had frontal osteitis, which allowed sampling and analysis of the purulent material.

Diagnostic methods

The diagnosis of African histoplasmosis relies on direct mycological examination, culture, and histopathological examination [4]. Although challenging, the culture of brain tissue and CSF provide the key evidence for the CNS infection [9]. Antigen detection sensitivity in cerebrospinal fluid (CSF) is reported in up to 67% in AIDS patients. Also, histopathology examination with Gomori methenamine silver or periodic acid-Schiff staining better visualizes the several large, thick-walled yeast cells seen mostly in histiocytic and giant cells [4].

Therapeutic options

CNS histoplasmosis can be a life-threatening condition. It is fatal if untreated. The therapeutic strategy is still controversial. Schestatsky et al. proposed a more aggressive induction phase with amphotericin B (40 mg/kg) for eight weeks [13]. One the other hand, the 2007 clinical practice guideline for histoplasmosis by Infectious Diseases Society of America (IDSA) advocated liposomal amphotericin B (5.0 mg/kg daily for a total of 175 mg/kg given over 4-6 weeks) followed by itraconazole (200 mg two or three times daily) for one year and until resolution of CSF abnormalities, including Histoplasma antigen levels [14]. Since the blood levels of itraconazole may vary widely in patients, a close serum monitoring along with renal and hepatic function are advised to optimize the treatment. The optimal serum level in AIDS patients on antiretroviral therapy (ART) is unknown. However, one should remain mindful of a possible immune reconstitution inflammatory syndrome (IRIS) in those patients during the phase of the treatment [10].

Although cases of brain or spinal cord lesions excision have been documented, the indication of surgery is still unclear, especially in patients with multifocal brain lesions. Moreover, successful cases of CNS histoplasmosis treatment by antifungal agents alone have been published [15].

## Conclusions

To the best of our knowledge, this case is the first case of cerebral localization of African histoplasmosis in the Ivory Coast and Africa. We believe this disease is still under-reported in our region. Since the advance in new immuno-suppressive drugs and the pandemic HIV infections, clinicians should expect to have more cases of CNS histoplasmosis. There is a need for personal protection equipment for individuals working in the poultry area. Further epidemiological analyses of national cases should lead to effective preventive actions by mapping of the possible reservoir of *Histoplasma capsulatum *var. *duboisii.*
